# Enhanced Fracture Toughness in Diamond/B_4_C Composites Through Residual-Stress-Induced Crack Deflection

**DOI:** 10.3390/ma19132708

**Published:** 2026-06-24

**Authors:** Yiyang Zhan, Zhengxin Li, Mu Qiao, Yujie Wang, Xuefei Fang, Yakun Lan, Guangli Zhu, Yuanmin Zou, Wenjie Yang, Chenyang Shi

**Affiliations:** 1 School of Materials Science and Engineering, Zhengzhou University, Zhengzhou 450001, China; 2 School of Materials Science and Engineering, Henan University of Technology, Zhengzhou 450001, China; 3 School of Chemical Engineering, Aalto University, 00076 Espoo, Finland

**Keywords:** diamond/B_4_C composite, HTHP, hardness, fracture toughness

## Abstract

Boron carbide (B_4_C) holds significant application potential in the fields of lightweight, high-hardness protective and high-end wear-resistant components due to its low density and exceptional hardness. However, its strong covalent bonding leads to low sintering activity and weak grain-boundary cohesion, resulting in high brittleness and crack sensitivity. These inherent properties make it difficult to achieve simultaneous full densification and toughness enhancement, severely limiting the reliability of B_4_C under complex service conditions. Although diamond is an attractive reinforcement because of its high elastic modulus and low coefficient of thermal expansion, the simultaneous realization of densification, graphitization suppression, and fracture-resistance improvement in diamond/B_4_C composites remains insufficiently understood. In this study, diamond particles were introduced into the B_4_C matrix and consolidated by rapid high-temperature and high-pressure (HTHP) sintering to synergistically promote densification and fracture toughening. The effects of sintering temperature and diamond content on phase evolution, densification, microstructure, and mechanical properties were systematically investigated, and the associated toughening mechanisms were analyzed. The results indicate that the hardness generally increases with rising sintering temperature and diamond content. The primary toughening mechanisms are identified as the pull-out of diamond particles and crack deflection induced by residual stresses generated during the cooling process. Although the composite with 20 wt.% diamond exhibits higher hardness, it also experiences severe macroscopic cracking. The composite with 10 wt.% diamond sintered at 1450 °C under 5.3 GPa for 4 min exhibits the optimal balance of properties, achieving a relative density of 98.85%, a Vickers hardness of 40.72 GPa, and a fracture toughness of 9.20 MPa·m^1/2^. This work confirms the effectiveness of combining diamond reinforcement with HTHP sintering in simultaneously achieving densification and toughening of B_4_C-based composites, providing a new pathway for developing high-performance lightweight protective ceramics.

## 1. Introduction

Boron carbide (B_4_C) is the third-hardest ceramic material after diamond and cubic boron nitride (CBN), with a density of 2.52 g/cm^3^ and a Vickers hardness of 29–32 GPa [1,2,3]. It has high hardness, high melting point, low density, high compressive strength, and excellent wear resistance, along with outstanding chemical stability and neutron absorption capacity. These properties make it highly suitable for applications in structural materials, the power industry, and nuclear engineering. B_4_C ceramic composites are used as lightweight armor materials, providing ballistic protection through fragmentation and energy absorption in the impact layer [4,5,6]. However, the strong covalent bonding structure and low self-diffusion coefficients of B_4_C ceramics makes sintering and densification of pure B_4_C ceramics challenging, leading to relatively low fracture toughness (2.16–2.52 MPa·m^1/2^) [7,8,9,10,11]. Improving the overall performance of B_4_C is essential for expanding its use in advanced protective systems and enabling the development of high-performance B_4_C-based composites.

To improve the mechanical properties of B_4_C ceramics, a variety of second-phase additives, including Al, Al_2_O_3_ SiC and TiB_2_, have been introduced during sintering [12,13,14]. Although TiB_2_ can improve fracture toughness, it often lowers hardness. Therefore, a key challenge is to identify a reinforcement that can simultaneously improve the hardness and fracture toughness of B_4_C while retaining its lightweight character. For example, Zhao et al. [15] fabricated B_4_C-Al_2_O_3_ composites by spark plasma sintering with Y_2_O_3_ as a sintering aid and achieved a relative density of 98.60%, whereas the hardness and fracture toughness remained limited to 23.75 GPa and 4.89 MPa·m^1/2^, respectively. Liu et al. [16] densified B_4_C under HTHP conditions of 4.5 GPa and 1300 °C for 5 min, achieving a density of 99.65% without abnormal grain growth. Liu et al. [17] also prepared TiB_2_-B_4_C composites at 5.0 GPa and 1500–2000 °C and found that both density and hardness increased with temperature, while Wang [18] reported a B_4_C-nano-TiB_2_ composite with a density of 99.95% prepared at 5.0 GPa and 1600 °C for 10 min.

Despite these advances, the hardness of many previously reported B4C-based composites remains unsatisfactory [14,19]. Introducing a second phase with both high hardness and high fracture resistance is therefore an attractive strategy for tailoring the mechanical performance of B_4_C. Diamond, with a hardness of 60–120 GPa and a relatively low density of 3.52 g·cm^−3^, is a particularly promising reinforcement [20,21]. In addition to improving hardness and wear resistance, diamond can also promote toughening through crack deflection, crack branching, and particle pull-out [22,23]. Moreover, because diamond has an extremely high elastic modulus and a much lower coefficient of thermal expansion than B_4_C, residual stresses may develop during cooling, further enhancing crack deflection and energy dissipation [24,25]. Moreover, to better understand the design of the present material, the constitution of the composite system and the role of each component should first be clarified. In the present composite system, B4C serves as the matrix phase, providing the lightweight and high-hardness framework of the material, whereas diamond acts as the reinforcing phase because of its ultrahigh hardness, high elastic modulus (1050 GPa), and low coefficient of thermal expansion (1.5 × 10^−6^/K) [26]. In addition, Si and Ti are introduced as reactive additive/binder phases to assist densification and interfacial bonding during high temperature sintering [27]. Specifically, Si can form a transient liquid phase and react with carbon to generate SiC, while Ti promotes the formation of Ti-containing interfacial reaction products such as TiB_2_ [28]. Therefore, the diamond/B_4_C composite studied here is not a simple two-phase ceramic system, but a reaction-assisted multiphase composite in which the matrix, reinforcement, and interfacial phases collectively govern the final densification behavior and mechanical performance.

The main processing routes for B_4_C ceramics include pressureless sintering, hot pressing, spark plasma sintering, and high-temperature high-pressure (HTHP) sintering [29,30,31,32,33,34]. Conventional densification methods generally require temperatures approaching 2000 °C, which can accelerate grain growth in B_4_C and increase the risk of diamond graphitization.

Although vacuum hot pressing and spark plasma sintering can achieve relative densities exceeding 90% at comparatively lower temperatures, they often require relatively long dwell times. Further increases in temperature may induce grain coarsening in B_4_C, thereby hindering densification and degrading mechanical performance [35]. Meanwhile, diamond is metastable at elevated temperatures and may undergo graphitization under inappropriate processing conditions, resulting in a loss of hardness. Consequently, the selection of a suitable sintering route is crucial. By contrast, HTHP sintering enables rapid densification within a short dwell time and can operate within the thermodynamic stability region of diamond, making it particularly suitable for fabricating diamond/B_4_C composites.

Diamond and graphite are two allotropes of carbon with distinct bonding structures and physical properties. Diamond possesses a three-dimensional sp^3^ covalent network, whereas graphite has a layered sp^2^-bonded structure. Under ambient pressure, diamond is metastable and may undergo graphitization at elevated temperatures, which would deteriorate its reinforcing effect. In contrast, under high-pressure conditions, the thermodynamic stability of diamond is significantly enhanced, and the diamond-to-graphite transformation can be effectively suppressed. This is important because preserving the sp^3^-bonded diamond phase helps retain its high hardness and reinforcing effect, whereas graphitization would weaken its contribution to densification and fracture resistance. Previous studies on B_4_C composites have mainly focused on TiB_2_, SiC, or oxide reinforcements, while high-pressure-sintered diamond/B_4_C ceramics have also been reported, most existing studies have primarily focused on densification and hardness improvement. By comparison, the influence of diamond incorporation on crack-propagation behavior and the associated toughening mechanisms, particularly those involving residual-stress-assisted crack deflection and particle pull-out, remains insufficiently understood. In addition, the role of the selected pressure–temperature window in preserving diamond stability while enabling densification has not been fully clarified. Therefore, the objectives of this work were to fabricate diamond/B_4_C composites by HTHP sintering within the pressure–temperature stability region of diamond, to systematically investigate the effects of sintering temperature and diamond content on phase evolution, densification, microstructure, and mechanical properties, and to clarify the toughening mechanisms associated with diamond incorporation.

## 2. Experimental

### 2.1. Materials

**Figure 1:**
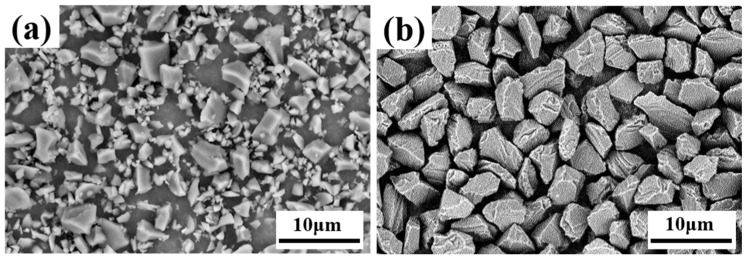
Microstructure of (**a**) B_4_C and (**b**) Diamond powders.

Commercial Diamond particles (W15 grade, 7–14 μm) were supplied by Henan Yalong Superhard Materials Co., Ltd. (Zhengzhou, China). The powder consisted of crushed diamond particles. Commercial B_4_C powder (2–4 μm, 99.9% purity) was purchased from Aladdin Biochemical Technology Co., Ltd. (Shanghai, China). Commercial Si powder (1–3 μm, 99.9% purity) and Ti powder (2–8 μm, 99.9% purity) were intentionally introduced as reactive additive/binder phases to promote densification and interfacial bonding during sintering. The contents of Si and Ti were fixed at 15 wt.% and 5 wt.%, respectively, based on preliminary formulation experiments carried out before the present study. These levels were retained throughout this work in order to provide consistent interfacial bonding and densification conditions, while allowing the effects of sintering temperature and diamond content to be evaluated more clearly. The reported diamond contents (0, 10, and 20 wt.%) were defined relative to the total powder batch. The nominal purity and particle-size ranges of all powders were based on supplier specifications. Therefore, the chemical composition information used in this work was based on supplier-reported purity, while the particle morphology and geometric characteristics of the starting powders were further examined by SEM, as shown in Figure 1.

### 2.2. Preparation Process of Diamond/B_4_C Composites

The powders were blended in a 100 mL stainless-steel ball-milling jar useing stainless steel balls with a ball-to-powder mass ratio of 4:1 and anhydrous ethanol as the milling medium. Planetary ball milling was carried out at 180 r/min for 16 h, with alternating forward and reverse rotation every 5 min. The slurry was then dried in a vacuum oven at 60 °C for 10 h and sieved to obtain a homogeneous mixed powder. The powder was loaded into a molybdenum cup and pre-compacted under an oil pressure of 20 MPa for 2 min. No additional organic binder was used during compaction; only the powder mixture containing B_4_C, diamond, Si, and Ti were employed. Here, Si acted as a reactive binder phase during sintering rather than as an added organic binder in the compaction step. After sintering, the samples were ground to remove the surface layer in contact with the molybdenum cup and then polished for characterization.

The mixed powders were sintered using the HTHP method at 5.3 GPa and temperatures of 1150, 1250, 1350, 1450, and 1550 °C. The heating time is 20 s, and the holding time was 4 min. No inert atmosphere was employed during the high-temperature hot-press sintering process, as the heating and holding times were very short. After sintering, the compacts were polished prior to characterization. The diamond contents were 0, 10, and 20 wt.%.

### 2.3. Characterization

After polishing, the phase constitution of the sintered diamond/B_4_C composites was analyzed by X-ray diffraction (XRD, MiniFlex 600, Rigaku Corporation, Japan, Cu Kα radiation, λ = 0.15406 nm; 40 kV, 100 mA; 2θ = 5–90°). The microstructure was examined by scanning electron microscopy (SEM, Phenom Pro X, FEI Company, Hillsboro, OR, USA).

The bulk density of the sintered diamond/B_4_C composites was measured by the Archimedes method and calculated using the following equation [36,37]:

(1) $$\rho_{1}=\frac{m_{1}}{m_{3}-m_{2}}\rho_{w}$$

In this equation, ρ_1_ is the density of the sintered sample (g/cm^3^), and ρ_w_ is the density of water at room temperature (g/cm^3^), where ρ_w_ = 1.0 g/cm^3^. Here, m_1_ is the dry mass of the sintered sample (g), m_2_ is the apparent mass of the sample suspended in water (g), and m_3_ is the wet mass of the sample measured in air (g).

The theoretical density (ρ) of the composite was calculated as follows [38]:

(2) $$\rho =\frac{100}{\frac{\omega_{1}}{\rho_{1}}+\frac{\omega_{2}}{\rho_{2}}+\frac{\omega_{3}}{\rho_{3}}+\frac{\omega_{4}}{\rho_{4}}}$$

In the equation, ρ is the theoretical density of the composite (g/cm^3^). The parameters ρ_1_, ρ_2_, ρ_3_, and ρ_4_ are the theoretical densities of B_4_C, diamond, Si, and Ti, respectively, and *w*_1_, *w*_2_, *w*_3_, and *w*_4_ are their corresponding mass fractions.

The relative density of the sample was calculated using the following equation:

(3) $$D=\frac{\rho_{1}}{\rho }\times 100\%$$

Diamond graphitization was evaluated by Raman spectrometer (LABRAM HR EVO, France) with an excitation wavelength of 532 nm. The thermal behavior of the mixed powders was examined by TG–DSC under an Ar atmosphere at a heating rate of 20 °C/min. Vickers hardness was measured and calculated using the following equation [39]. This method was selected because it is widely used for dense ceramic composites and enables direct comparison with previously reported B_4_C-based materials.

(4) $$HV=\frac{1.854P}{d^{2}}$$

In this equation, P represents the applied load (N), and d is the average indentation diagonal length (mm).

The fracture toughness, K_IC_, was estimated from the indentation crack length using the following equation [40,41]:

(5) $$K_{IC}=0.016HV\times {\left(\frac{c}{d}\right)}^{\frac{3}{2}}\times d^{\frac{1}{2}}$$

In this equation, d is the average indentation diagonal length (mm), a is the half-diagonal length (mm), c is the corresponding crack length (mm), where c = l + a. Here, HV denotes the Vickers hardness (kgf/mm^2^ or N/mm^2^).

## 3. Results and Discussion

The microstructures of the starting B_4_C and diamond powders are shown in Figure 1. The B_4_C powder consisted of uniformly distributed particles with a size of 1–3 μm. The crushed diamond particles exhibited rough surfaces and particle sizes of 7–14 μm, which are favorable for enhancing interfacial bonding with the matrix.

**Figure 2:**
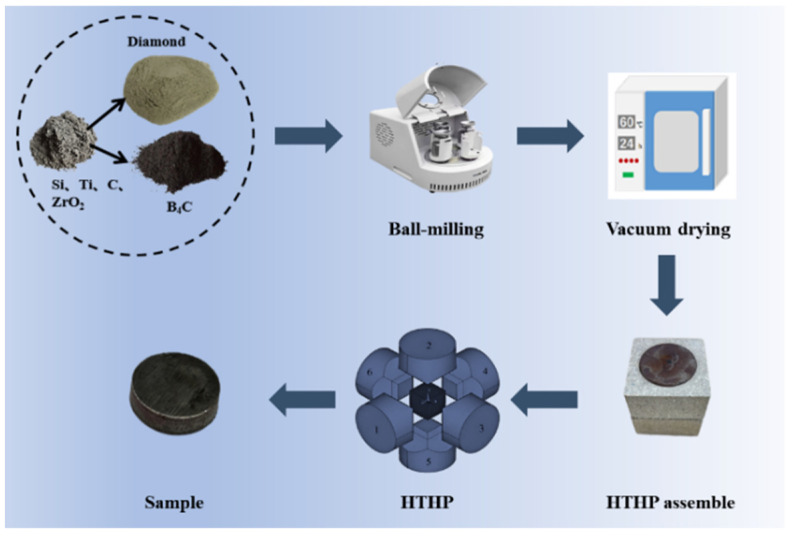
Schematic illustration of the HTHP sintering process.

After pre-compression, the blocks are sintered at high temperature and pressure in cubic press. The sintering process of the composite sample is illustrated in Figure 2.

The densities of the composites sintered at 1150, 1250, 1350, 1450, and 1550 °C are summarized in Table 1. The results indicate that high-temperature high-pressure sintering at 5.3 GPa and 1450 °C enables the fabrication of diamond/B_4_C composites with near-full densification. Because diamond possesses a relatively high intrinsic density, the bulk density of the diamond/B_4_C composites gradually increases with increasing diamond content. The specimen sintered at 1550 °C exhibits the highest measured density. However, such an elevated temperature may induce partial graphitization of diamond. The formation of graphite at grain boundaries can facilitate grain-boundary diffusion and thereby accelerate the densification process [42,43,44]. Nevertheless, graphitized regions on the diamond surface possess significantly lower hardness than diamond, which deteriorates the mechanical performance of the composite.

**Figure 3:**
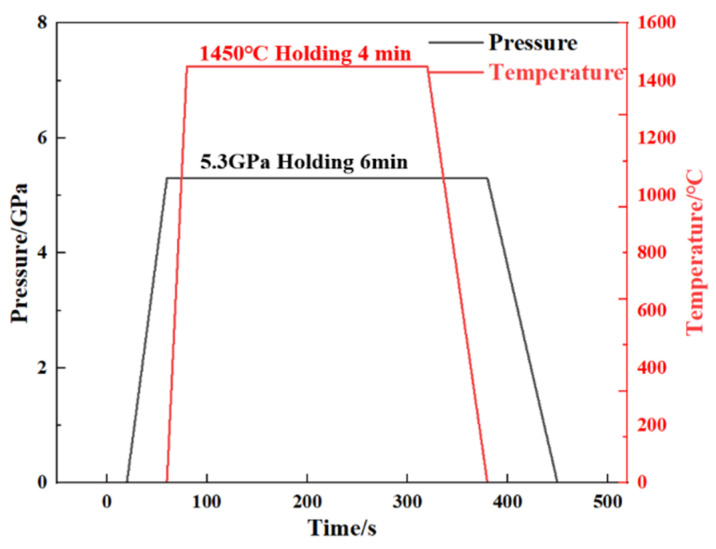
Temperature–pressure profile for HTHP sintering at 1450 °C.

Considering both densification behavior and phase stability, the composite samples sintered at 1450 °C exhibit the most balanced overall performance. The corresponding temperature–pressure processing diagram for sintering at 1450 °C is presented in Figure 3. Based on these results, the effect of diamond content on the composite properties was further investigated by varying the diamond addition to 0 wt.%, 10 wt.%, and 20 wt.%.

**Figure 4:**
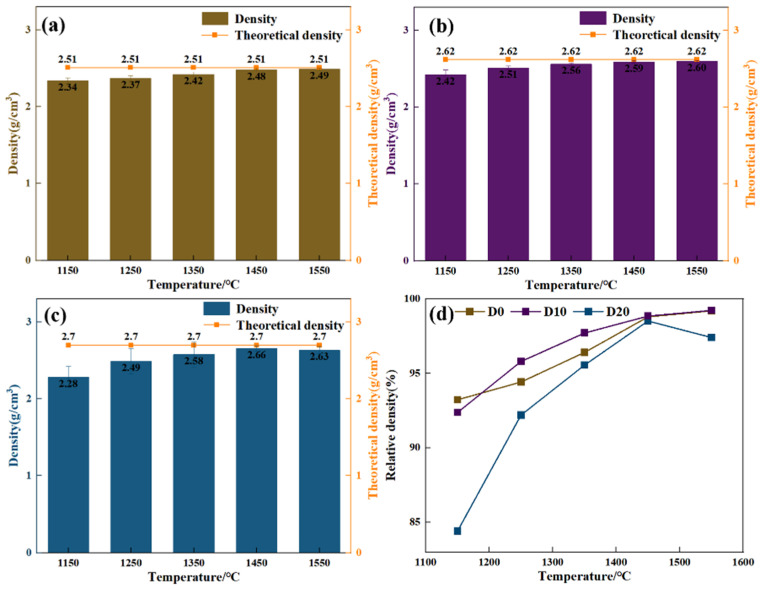
Density of Diamond/B_4_C specimens as a function of Diamond content at different temperature: (**a**) 0 wt%; (**b**) 10 wt%; (**c**) 20 wt%; (**d**) Comparison of Density after Sintering with Different Diamond Contents.

The density and relative density of the composites as functions of sintering temperature and diamond content are presented in Figure 4. The results demonstrate that both the bulk and relative densities increase with higher sintering temperatures and diamond content. Under ultrahigh-pressure conditions, particle rearrangement is significantly enhanced. This process reduces intergranular spacing, eliminates voids, and promotes close grain-to-grain contact, all of which accelerate densification. Because Si was introduced as a reactive additive, the formation of a Si-rich liquid phase plays an important role in the sintering process. At lower temperatures, however, the amount of liquid phase remains limited, which restricts interparticle bonding and densification. As the temperature rises, liquid-phase sintering becomes the dominant mechanism, facilitating particle rearrangement and promoting interfacial wetting. This process improves densification and reduces porosity. The ultrahigh-pressure environment also plays a crucial role in stabilizing the diamond phase. Due to the short sintering time and high pressure, the temperature required for graphitization is effectively increased, thereby helping to preserve the hardness of diamond and maintain the integrity of the reinforcement phase. As diamond content increases, the composite density rises due to the higher intrinsic density of diamond. Although the 20 wt.% sample exhibited a higher bulk density because of the higher intrinsic density of diamond, the 10 wt.% sample showed the highest relative density, reaching 98.85% with a bulk density of 2.59 g/cm^3^ after sintering at 1450 °C. This relative density is close to the 98.60% reported for SPS-sintered B_4_C-Al_2_O_3_ composites [15], although lower than the 99.65% of HTHP-sintered monolithic B_4_C [16] and the 99.95% of HTHP-sintered B_4_C-nano-TiB_2_ composites [18]. Although the optimum sample approached full densification, limited residual porosity may still remain locally, particularly near interfaces or incompletely bonded regions. Under static loading, such porosity may act as a stress concentrator and reduce the mechanical reliability of the composite. However, under impact-dominated loading, such as in lightweight protective ceramics, a limited amount of finely distributed residual porosity may contribute to local stress relaxation and energy dissipation by promoting crack deflection or branching. Nevertheless, excessive porosity would be detrimental to both densification and structural integrity. Considering that the present system contains a metastable diamond reinforcing phase and was processed under a relatively short sintering cycle, these results still indicate that the selected HTHP window is effective for rapid densification.

**Figure 5:**
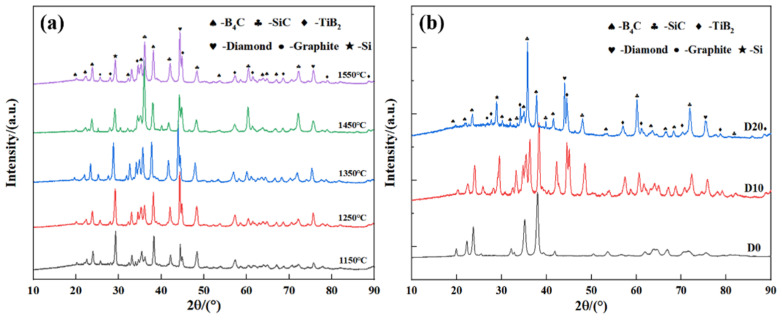
XRD patterns of composite materials (**a**) at different temperatures (**b**) with varying diamond content.

The X-ray diffraction (XRD) patterns of the composites sintered at different temperatures and diamond contents are shown in Figure 5. Distinct B_4_C and diamond peaks were observed, indicating that no detectable reaction occurred between diamond and B_4_C and that diamond did not undergo graphitization. With increasing temperature, the SiC peaks became progressively more intense. As the temperature approaches the melting point of Si (around 1414 °C), molten Si gradually infiltrates the compact, leading to a more complete reaction. Weak residual Si peaks were observed at approximately 2θ = 25.2° and 32.7°. This is attributed to the rapid heating and short dwell time characteristic of HTHP sintering: densification is accelerated under high temperature and pressure, while the short dwell time may leave a small fraction of Si unreacted and redistributed to the sample surface. No distinct graphite peak at 2θ ≈ 26.6° was detected below 1450 °C, suggesting that although diamond is metastable at elevated temperatures and can transform to graphite, graphitization was limited under ultrahigh pressure and the short sintering time. The diamond peaks increased in intensity with increasing diamond content, whereas the B_4_C peaks weakened. It is well known that diamond is metastable under ambient pressure and may begin to graphitize at temperatures above 700 °C; therefore, conventional hot pressing and SPS routes involving diamond must address this issue. In contrast, high-pressure sintering can operate within the thermodynamically stable region of diamond, thereby suppressing graphitization and making it well suited for fabricating diamond/B_4_C composites. At 20 wt.% diamond, the B_4_C peaks became weak while the SiC peaks increased markedly. This trend is attributed to the increased carbon supply at higher diamond contents, which promotes SiC formation through reaction with Si. As SiC becomes more prevalent, the overall hardness of the composite may decrease.

The following reactions occurred in this experiment [45]:

Si(l) + C(s, diamond surface) → SiC(s)(6)

5Ti(s) + B_4_C(s) → 2TiB_2_(s) + TiC(s) (7)

During HTHP, silicon forms a transient liquid phase that reacts with free carbon originating from both the diamond surface and boron carbide, leading to the formation of SiC. The thermodynamic feasibility of this reaction pathway as well as the identity of the reaction products and their equilibrium relationships can be rationalized using the Si-C binary phase diagram under pressures of 0.1–8 GPa reported [46]. A key feature of the Si-C system under elevated pressure is that SiC represents the only thermodynamically stable compound throughout the entire temperature range. Consequently, when silicon reacts with carbon originating from diamond (C) or boron carbide (B_4_C), the reaction pathway is highly constrained, yielding SiC as the sole stable product while effectively suppressing the formation of other Si-C intermediate phases. At atmospheric pressure, the Si-C system exhibits a eutectic reaction near 1400 °C in the Si-rich region, enabling the formation of a transient liquid phase during sintering. When the temperature reaches 1450 °C, silicon can therefore generate a liquid phase through the eutectic reaction even without reaching its melting point. At this temperature, the Si-rich region falls entirely within the liquid phase (L). The application of high pressure (4–8 GPa) further expands the stability region of the liquid phase and lowers the temperature required for liquid formation, enabling a substantial amount of liquid Si to appear during the early stages of sintering.

The presence of this liquid phase plays a critical role in the densification process. It enhances particle wetting, promotes intimate interfacial contact, and provides an efficient medium for mass transport. At the same time, elevated pressure significantly increases the solubility of carbon in liquid silicon, allowing additional carbon atoms to dissolve from the surfaces of diamond and B_4_C particles. Once dissolved, these atoms diffuse rapidly through the liquid phase. Diffusion distances decrease, intergranular pores are compressed, and interfacial reactions proceed more efficiently. As a consequence, a continuous and dense SiC reaction layer gradually forms along the diamond/B_4_C interfaces, strengthening the bonding between the phases and contributing to the development of a compact microstructure. High pressure also plays another essential role. It stabilizes the diamond phase (D) and suppresses its high-temperature transformation into graphite. This stabilization preserves the structural integrity of the diamond reinforcement during sintering. Under the combined conditions of 1450 °C and 4–8 GPa, SiC remains thermodynamically stable and coexists with diamond within a stable two-phase region (SiC + D).

Within the Ti-B-C ternary system, TiB_2_ represents the dominant thermodynamically stable boride phase in the low-Ti, B-C-rich compositional region [47]. Under such conditions, the formation of other Ti-B binary phases is thermodynamically unfavorable, making TiB_2_ the principal reaction product involving titanium and boron. Energetically, the Ti-B bond possesses a substantially higher bonding energy than the Ti-C bond. Consequently, Ti atoms preferentially react with boron atoms originating from B_4_C. This preferential reaction pathway promotes the formation of TiB_2_, while only a limited amount of TiC is generated as a secondary byproduct. In contrast, the carbon atoms on the diamond surface adopt a highly stable sp^3^-hybridized configuration. The strong covalent bonding within this structure significantly reduces their chemical reactivity toward Ti, thereby preserving the structural integrity of the diamond phase during the reaction process. The TiB_2_ phase formed during sintering is primarily distributed along B_4_C grain boundaries and at diamond-B_4_C interfaces. Such a spatial distribution is beneficial for microstructural regulation. TiB_2_ particles exert a grain-boundary pinning effect that inhibits grain growth, while also promoting crack deflection during fracture propagation.

**Figure 6:**
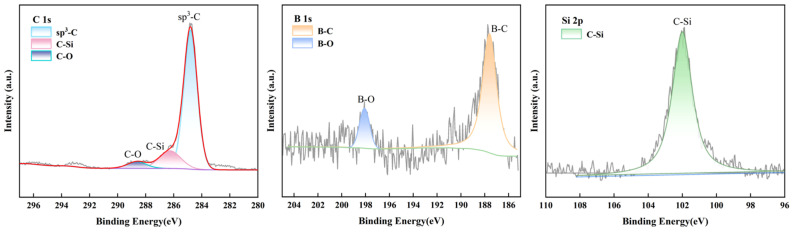
XPS spectra of C 1s, B 1s, and Si 2p core levels.

To elucidate the chemical bonding characteristics at the diamond-B_4_C interface, X-ray photoelectron spectroscopy (XPS) analyses on the C 1s, B 1s, and Si 2p core levels, as shown in Figure 6. The C 1s spectrum exhibits a dominant peak at 284.8 eV, corresponding to the sp^3^-hybridized C-C bonds of diamond. The sharp and symmetric peak shape and the absence of a noticeable sp^2^-C component indicate that diamond retains its sp^3^ bonding structure after HTHP sintering, suggesting that graphitization is effectively suppressed. A weak peak at 286.5 eV is assigned to C-Si bonds, characteristic of SiC, providing evidence of interfacial reactions between Si and carbon during sintering. In addition, a minor peak near 289 eV is attributed to surface C-O species, which likely originate from slight surface oxidation after exposure to air [48]. The B 1s spectrum shows a dominant peak at 188 eV, corresponding to B-C bonds in B_4_C, confirming the structural stability of the B_4_C matrix during sintering. A secondary peak at 198 eV is assigned to B-O bonding, which is attributed to minor surface oxidation occurring during sample handling or air exposure. The Si 2p spectrum exhibits a characteristic peak corresponding to C-Si bonding, further confirming the formation of SiC at the diamond-matrix interface. These results collectively demonstrate that interfacial reactions between Si and carbon occur during HTHP sintering while the diamond phase largely preserves its sp^3^ structure.

**Figure 7:**
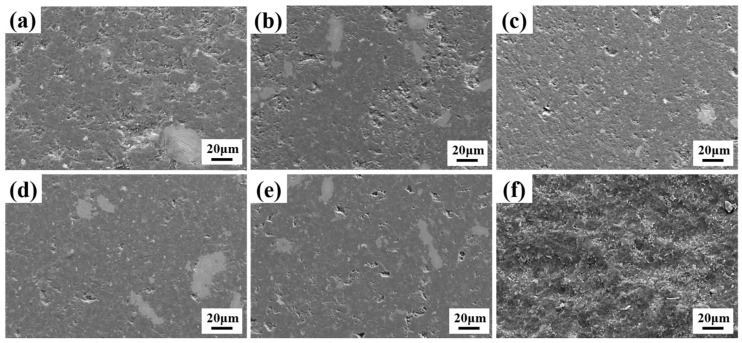
Polished surfaces of sintered diamond/B_4_C composites at different temperatures: (**a**) 1150 °C; (**b**) 1250 °C; (**c**) 1350 °C; (**d**) 1450 °C; (**e**) 1550 °C; (**f**) cross-section at 1450 °C.

Microstructural images of sintered Diamond-B_4_C composite samples at different temperatures are presented in Figure 7a–e, and a cross-sectional view of the specimen sintered at 1450 °C is shown in Figure 7f. The results show that the density of the samples increases gradually with rising sintering temperature. At 1450 °C, the composite exhibits the highest density, and the cross-sectional microstructure shows diamond pull-out features and no visible voids. At the lower sintering temperature, the observed pores may be associated with incomplete densification, partial surface graphitization, and interfacial thermal-stress concentration. For specimens sintered outside the optimum window, this interpretation should be regarded as a plausible inference rather than direct experimental proof [49]. The large mismatch in the coefficients of thermal expansion between diamond and B_4_C creates differential thermal strain during heating, leading to interfacial thermal stresses [50,51]. Under high-pressure conditions, this mismatch can intensify stress concentration at the interface, further promoting pore formation.

**Figure 8:**
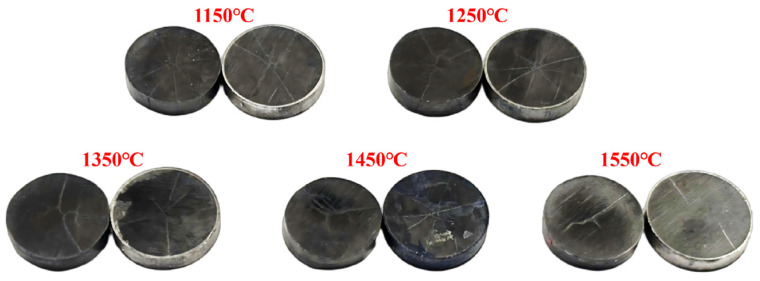
Photographs of specimens sintered at 1150, 1250, 1350, 1450, and 1550 °C.

The physical photograph of the samples are shown in Figure 8. Multiple distinct voids are observed in the samples sintered at 1550 °C. At such an excessively high temperature, these defects may be associated with partial surface graphitization or intensified interfacial stress effects, both of which would hinder densification and lead to a lower composite density.

**Figure 9:**
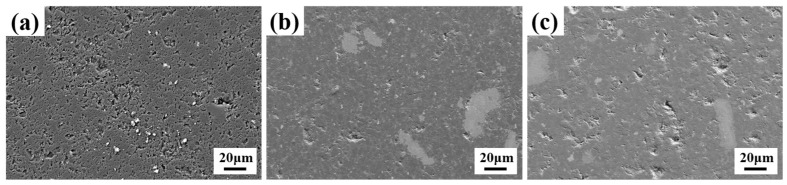
Polished surfaces of composites sintered at 1450 °C with different diamond contents: (**a**) 0 wt.%; (**b**) 10 wt.%; (**c**) 20 wt.%.

Microstructural images of composite samples at different diamond contents are displayed in Figure 9a–c. The addition of diamond enhances the densification by promoting interfacial interactions between the constituent phases under high temperature and pressure conditions. Surface carbon from the diamond phase can react with binder elements such as Si and Ti, thereby facilitating interfacial bonding and contributing to the sintering process. However, the specimen with 20 wt.% diamond content exhibits significant macroscopic cracking, which becomes more pronounced at higher sintering temperatures. This behavior is primarily attributed to the large thermal expansion mismatch between diamond and B_4_C. As diamond content increases, the number of particle-matrix interfaces also increases, leading to higher thermal stress accumulation and intensified interfacial stress concentration, which ultimately results in extensive cracking. Simultaneously, excessive diamond content can promote agglomeration, which reduces dispersion homogeneity [52] and further compromises both densification and mechanical performance. Therefore, an optimal diamond content is crucial for balancing densification and mechanical integrity.

**Figure 10:**
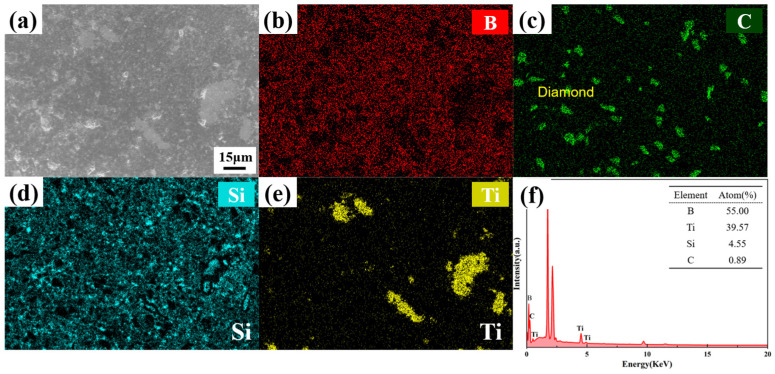
EDS of the polished surface of the composite sintered at 1450 °C with 10 wt.% diamond: (**a**) EDS analysis area; (**b**) B-K; (**c**) C-K; (**d**) Si-K; (**e**) Ti-K; (**f**) elemental composition corresponding to (**a**).

The elemental maps and compositional distribution of the W15 diamond/B_4_C composite sintered at 1450 °C are presented in Figure 10a–f. The diamond particles are uniformly distributed throughout the B_4_C matrix, resulting in a dense microstructure with a homogeneous distribution of both binder phase and diamond. The dark regions correspond to the areas of diamond/B_4_C interfacial bonding, while the gray regions are binder-enriched zones containing Si and Ti, along with in situ reaction products. Consistent with the XRD results, most of the Si is consumed during sintering, reacting with B_4_C and carbon to from SiC. This SiC phase is uniformly distributed throughout the composite. These observations further validate the phase distribution and the extent of interfacial reactions that occur within the composite structure.

**Figure 11:**
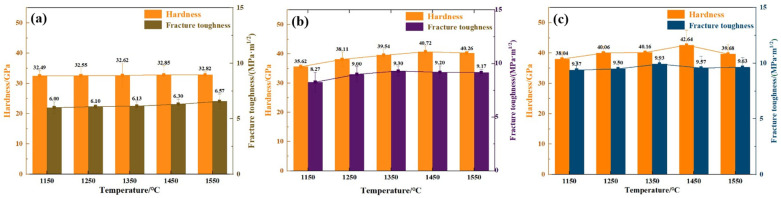
Hardness and Fracture Toughness of diamond/B_4_C composites Sintered at Different Temperatures: (**a**) 0 wt.% diamond; (**b**) 10 wt.% diamond; (**c**) 20 wt.% diamond.

The hardness and fracture toughness of diamond/B_4_C composites are shown in Figure 11. The fracture toughness was estimated from the cracks generated after Vickers indentation. In the absence of diamond, the monolithic B_4_C ceramic exhibited a hardness of 32.85 GPa and a fracture toughness of 6.30 MPa·m^1/2^. With diamond addition, both hardness and toughness increased, reaching 40.72 GPa and 9.20 MPa·m^1/2^ at 10 wt.% diamond. At 20 wt.% diamond and 1450 °C, the hardness and fracture toughness further increased to 42.64 GPa and 9.57 MPa·m^1/2^. This increase is likely due to a denser diamond distribution within the composite. During Vickers testing, the Vickers impression is more likely to overlap with large diamond particles at higher diamond contents, which may lead to locally elevated hardness values. Compared with monolithic B_4_C, diamond addition significantly improved both hardness and fracture toughness. The 10 wt.% diamond composite exhibited a much more favorable combination of mechanical properties than SPS-sintered B_4_C-Al_2_O_3_ composites reported by Lyu et al. [15], which showed a hardness of 23.75 GPa and a fracture toughness of 4.89 MPa·m^1/2^. In addition, Liu et al. [17] reported that the density and hardness of HTHP-sintered TiB_2_-B_4_C composites increased with temperature, whereas the present results further indicate that diamond reinforcement is highly effective for enhancing hardness while maintaining a high level of fracture resistance in B_4_C-based composites. The hardness achieved in the present work is also comparable to that reported for high-pressure-sintered diamond/B_4_C ceramics [20]. Although the 20 wt.% diamond sample exhibited slightly higher hardness and indentation-derived fracture toughness, obvious macroscopic cracking was observed, suggesting reduced structural integrity. Therefore, the 10 wt.% diamond composite was considered to offer the best overall balance between mechanical performance and structural stability.

**Figure 12:**
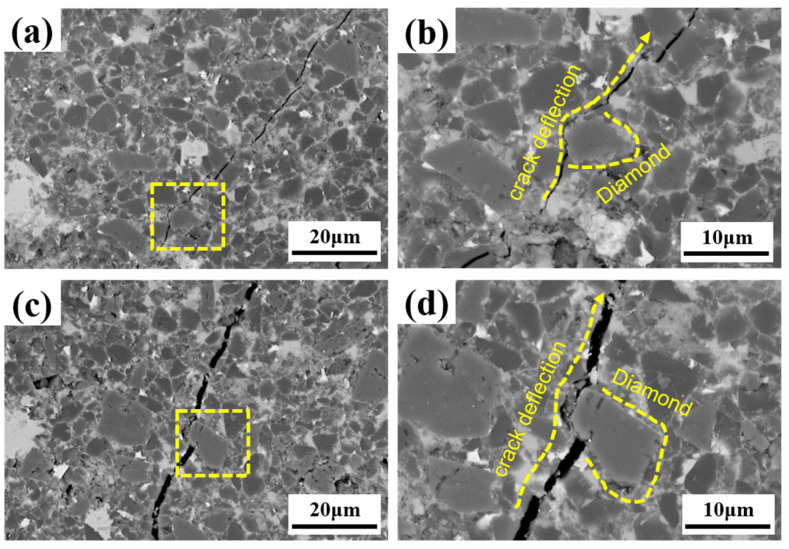
SEM micrographs of cracks on the polished surface of diamond/B_4_C composite ceramics containing 10 wt.% diamond. (**b**) is a higher-magnification view of (**a**), and (**d**) is a higher-magnification view of (**c**).

Crack propagation in the composite containing 10 wt.% diamond sintered at 1450 °C is illustrated in Figure 12. Diamond particles are uniformly distributed in the B_4_C matrix, reducing porosity. Monolithic B_4_C typically fails by intergranular brittle fracture. In the present composite, intergranular fracture remains dominant, with cracks propagating around and across diamond particles, indicating strong diamond/B_4_C interfacial bonding. Overall, diamond addition does not fundamentally change the intrinsic fracture mode of the B_4_C matrix. The large mismatch in thermal expansion coefficients between diamond and B_4_C generates residual stresses at particle-matrix and grain boundaries during cooling [53,54,55]. The residual stress in the composite is estimated using the following equation [56]:

$$\sigma i=\frac{Ei}{1-v}(\alpha i-\sum \omega_{j}\alpha_{j})∆T$$

where E is the elastic modulus, α is the coefficient of thermal expansion, ΔT is the temperature change, and ν is the Poisson’s ratio. In this calculation, the residual stress was estimated using a thermal-expansion-mismatch model under the assumptions of isotropic linear elasticity, uniform cooling, and effective interfacial constraint between the diamond particle and the B_4_C matrix. For diamond, E = 1050 GPa, α = 1.2 × 10^−6^/K, and ν = 0.20; for B_4_C, E = 450 GPa, α = 4.5 × 10^−6^/K, and ν = 0.17. The temperature change ΔT was defined as the difference between the sintering temperature and room temperature (25 °C). Under these assumptions, the calculated residual stresses were approximately −5.38 GPa in diamond and 0.33 GPa in the B_4_C matrix. When cracks propagate toward the diamond particle boundary, the compressive stress within the diamond tends to inhibit direct crack penetration and promotes crack deflection along the diamond/B_4_C interface (arrowed). This increases the effective crack length, promoting energy dissipation. Thus, the toughening of diamond/B_4_C composites is primarily governed by residual-stress-assisted crack deflection and intergranular fracture, with contributions from diamond pull-out and energy absorption [57]. These mechanisms hinder crack propagation and enhance fracture toughness. The fracture toughness values reported here were estimated using the indentation crack method, which may introduce uncertainties compared to standard methods like single-edge notched beam (SENB) or chevron-notched beam (CNB) tests. Therefore, these values should be seen as comparative indicators of the toughening effect of diamond addition, rather than absolute fracture toughness values.

**Figure 13:**
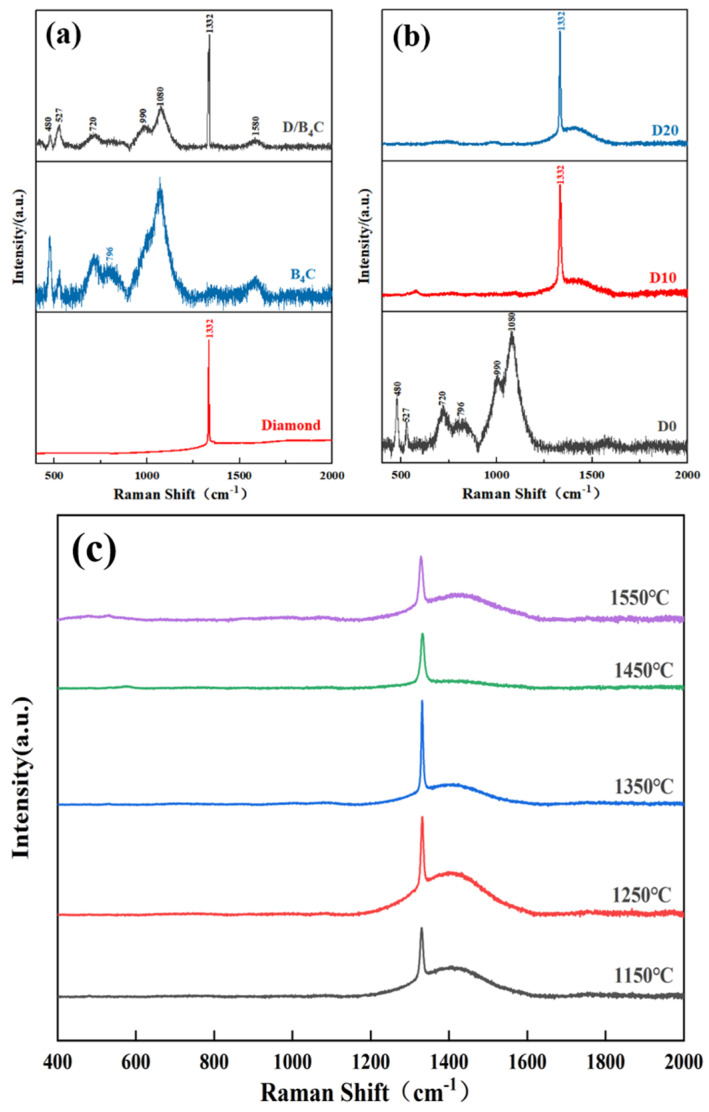
Raman spectra of HTHP-prepared composites: (**a**) composite material; (**b**) Raman spectra of diamond at different diamond contents; (**c**) Raman spectra of diamond at different sintering temperatures.

The Raman spectrum of the HTHP-prepared diamond/B_4_C composite (1450 °C, 10 wt.% diamond) is shown in Figure 13. In the sample without diamond addition, the characteristic Raman bands are primarily assigned to B_4_C (480, 527, 720, 990, and 1080 cm^−1^) and SiC (796 cm^−1^). After diamond incorporation, slight peak shifts are observed, likely due to residual stresses in the composite and/or the formation of new crystalline phases during HTHP sintering. Spectra from diamond-rich regions show the characteristic diamond peak at 1332 cm^−1^. No graphite-related peaks are detected, and the diamond peak remains dominant, indicating that diamond graphitization is effectively suppressed under the elected optimum HTHP conditions. This absence of a graphitic signal aligns with the XPS results. Although these shifts were not used for direct quantitative stress analysis, they are consistent with the calculated stress state and with the observed crack deflection around diamond particles.

## 4. Conclusions

In this work, dense diamond/B_4_C composites with high hardness and improved fracture resistance were successfully fabricated by combining diamond-particle reinforcement with rapid high-temperature and high-pressure (HTHP) sintering. The effects of sintering temperature and diamond content on phase evolution, densification, microstructure, and mechanical properties were systematically investigated. The results showed that HTHP sintering within the selected pressure–temperature window effectively suppressed diamond-to-graphite transformation while promoting the densification of B_4_C-based composites. During sintering, interfacial reactions between diamond and the Si/Ti binder produced minor amounts of SiC and TiB_2_, which contributed to grain-growth suppression and densification. The improved fracture resistance was mainly attributed to residual-stress-assisted mechanisms, including diamond pull-out and crack deflection, which increased crack-path tortuosity and enhanced fracture energy dissipation. Although the composite with 20 wt.% diamond exhibited slightly higher hardness and indentation-derived fracture toughness, obvious macroscopic cracking was observed, suggesting reduced structural integrity. Therefore, the composite with 10 wt.% diamond sintered at 1450 °C under 5.3 GPa for 4 min showed the best overall performance, with a relative density of 98.85%, a Vickers hardness of 40.72 GPa, and a fracture toughness of 9.20 MPa·m^1/2^. Overall, this study achieved the objectives of fabricating dense diamond/B_4_C composites under HTHP conditions, systematically clarifying the effects of sintering temperature and diamond content on phase evolution, densification, microstructure, and mechanical properties, and identifying residual-stress-assisted crack deflection together with diamond pull-out as the main toughening mechanisms.

## Figures and Tables

**Table 1:** Density of monolithic Diamond/B_4_C sintered from 1150 °C to 1550 °C.

Temperature (°C)	0 wt%	10 wt%	20 wt%
1150	2.34	2.42	2.28
1250	2.37	2.51	2.49
1350	2.42	2.56	2.58
1450	2.48	2.59	2.66
1550	2.49	2.60	2.63

## Data Availability

The original contributions presented in this study are included in the article. Further inquiries can be directed to the corresponding authors.
